# Aging Reduces the Efficiency of Parafoveal Lexical Activation During Chinese Sentence Reading

**DOI:** 10.3390/jemr19020035

**Published:** 2026-04-01

**Authors:** Yiu-Kei Tsang, Ming Yan, Jinger Pan

**Affiliations:** 1 Department of Education and Psychology, Hong Kong Baptist University, Hong Kong, China; 2 Department of Psychology, University of Macau, Macau, China; mingyan@um.edu.mo; 3 Center for Cognitive and Brain Sciences, University of Macau, Macau, China; 4 Department of Psychology, The Education University of Hong Kong, Hong Kong, China; jpan@eduhk.hk

**Keywords:** cognitive aging, parafoveal processing, preview benefits, eye movement, Chinese reading

## Abstract

This study utilized the gaze-contingent boundary paradigm to examine age-related changes in parafoveal processing during Chinese sentence reading. A community sample of 65 older readers and 68 younger readers from Hong Kong read 130 sentences while their eye movements were recorded. In each sentence, an invisible boundary was placed just before a critical target word. Before the readers’ eye gaze crossed the boundary, a parafoveal preview was presented in the position of the target word. The preview could be identical, orthographically related, phonologically related, semantically related, or unrelated to the first character of the target word. Once the eye gaze passed the boundary, the preview characters changed to the target. For the younger readers, the related parafoveal previews facilitated the subsequent foveal processing of the target compared to the unrelated previews across early and late eye movement measures. In contrast, the older readers demonstrated a reduced identical preview benefit in early eye movement measures. They also showed benefits in other preview conditions only in later measures. These results suggest that older Chinese readers can extract linguistic information from parafoveal vision despite reduced visual acuity. However, the efficiency of parafoveal processing is reduced, potentially due to slower processing speed and less efficient spreading activation within the lexical network.

## 1. Introduction

Reading is an intricate mental activity that requires the coordination of multiple cognitive processes [1]. Traditionally, research on reading development has focused on reading acquisition in children [2]. However, reading development is a lifelong process. As population aging is becoming a global concern, there is growing interest in studying reading development in later life [3], which can contribute to the theoretical understanding of the reading process in older readers and has practical implications for improving reading efficiency among the elderly. It is known that older adults suffer from declines in cognitive functions and visual acuity, leading to slower reading [3,4]. Yet, little is known about whether aging impacts the processing of different types of linguistic information differentially, while such information is essential to incorporate a developmentally valid word recognition module into existing models [5]. To fill in the research gap, this study focused on a specific aspect of reading among older readers, namely the efficiency of their parafoveal processing. By comparing their performance with that of younger readers in an eye-tracking experiment with the gaze-contingent boundary paradigm [6], we tested whether older readers could extract different types of linguistic information from parafoveal vision.

### 1.1. Cognitive Aging and Reading

While crystallized intelligence remains relatively well-preserved or even improves with age, age-related declines in visual ability and processing speed are well-documented [7,8]. Despite these challenges, reading remains a major daily activity in acquiring new information for many older adults. The accumulation of reading experiences may lead to compensatory strategies to maintain reading efficiency [9]. How aging affects reading is dependent on the complex interplay between these factors. Empirically, older adults have demonstrated slower word recognition speed and reduced reading rate compared to younger adults [10,11], consistent with the decline in processing speed. However, their vocabulary size and word recognition accuracy remain comparable to or even surpass those of younger adults [11,12], aligning with the preservation of crystallized intelligence in the aging population. Reading comprehension is also relatively intact, at least for narrative texts [4,9].

In addition to paper-and-pencil tests or simple reaction time experiments, researchers have also utilized eye-tracking to examine aging effects on reading, which has the advantage of providing fine-grained details about online cognitive processing. The results reveal further differential aging effects on reading subprocesses. For example, older adults appear to be at least as sensitive to word predictability during reading as younger adults, suggesting intact top-down semantic modulation [4,13]. Yet, they show greater difficulty in processing low-frequency words or texts printed in difficult fonts, indicating that their bottom-up visual and lexical processing are more disrupted [4,14,15]. Older readers also tend to fixate closer to word beginnings, which is a suboptimal position for efficient word recognition [16,17], which may reflect problems in their oculomotor control.

Collectively, the available findings indicate that while specific subprocesses of reading (e.g., visual perception) decline with age, older readers are often able to extract word meanings and maintain overall satisfactory comprehension. As a highly practiced skill, the “selective optimization with compensation” framework of cognitive aging assumes that older readers can optimize their processing and develop compensatory strategies to maintain their overall reading performance, such as relying more on prior knowledge [9,18]. Some eye-tracking studies have provided evidence for this claim. For instance, Laubrock et al. [19] re-analyzed the Potsdam Sentence Corpus [14] and found increased word skipping and more frequent regressions among older readers. Rayner et al. [4] obtained similar results in English sentence reading, which led the authors to propose that older readers tend to adopt a “risky” reading strategy to compensate for the general slower processing speed. Specifically, the authors argued that older readers are more likely to guess the upcoming words based on “partial parafoveal information” (p. 457, [4]) and their capacity of “having more reading experience” (p. 458, [4]), which results in more skipping and potentially faster reading speed. However, when the guesses turn out to be incorrect, the readers need to regress back to earlier parts of the sentence for reprocessing and clarification, offsetting the benefits in reading speed while maintaining the overall high level of comprehension accuracy.

On the other hand, in a recent meta-analysis, Zhang et al. [20] showed that older readers exhibited higher skipping rates than young readers only in reading alphabetic scripts. A similar pattern was not observed in older Chinese readers (also see [18]). Moreover, words of higher frequency or predictability did not increase skipping rate as the risky reading strategy predicted. These findings challenge the universality of the risky reading strategy in older readers and call for further research. In this study, we examined whether providing different types of information in the parafovea would differentially affect the processing in older and younger readers.

### 1.2. Aging and Parafoveal Processing

Given the age-related visual decline, it seems reasonable to expect less efficient parafoveal processing in older readers. Xie et al. [21] investigated the visual span size of younger and older readers using a trigram task, where sets of three letters were displayed at varying distances from the fixation point (as measured in number of letters). Their results showed that older readers’ average span size was 1.2 letters smaller than that of younger readers, supporting less efficient parafoveal processing. In Rayner et al. [22], a moving-window paradigm [23] was adopted, creating a visual “window” displaying text contingent on the current fixation location, while text outside this window was masked. The window was synchronized with the readers’ eye gaze, such that a new window was created after each saccade to provide new information around the new fixation point. Moreover, the amount of information available to the readers in each fixation, that is, the size of the window, was under experimental manipulation. Results indicated that the older readers could only obtain useful linguistic information one word to the right of the fixation compared to two words for young readers, indicating a smaller perceptual span, which is the region from which useful linguistic information can be extracted in one fixation, among older readers.

However, the efficiency of parafoveal processing also depends on attentional factors [9,24]. Using SWIFT simulation to recover experimental age differences in fixation duration and probability, Laubrock et al. [19] did not obtain the predicted reduction in the perceptual span, as suggested by Rayner et al. [22]. Instead, their simulation produced a perceptual span with greater rightward asymmetry. Because the perceptual span reflects mostly attentional influences, the findings suggested that older readers may allocate more attentional resources to process upcoming words parafoveally, potentially to “counteract influences of the relative loss in visual acuity” (p. 881, [19]). Similarly, Risse and Kliegl [24] found that both younger and older German readers were able to extract information two words from the right of the fixation (*N* + 2 position), differing from Rayner et al.’s [22] results in English. Xie et al. [15,25] also reported comparable perceptual span sizes in older and younger readers in two Chinese moving window experiments. Similarly, in a group of French–English bilinguals, Whitford and Titone [26] observed similar span sizes (14 letters to the right of fixation) across age groups, although reading rates were in general slower in L2 and older readers. Furthermore, better executive control was associated with a larger span size among older adults, but not younger ones. This led the authors to propose that older adults adopt a reading strategy that prioritizes parafoveal processing, which may allow them to extract useful linguistic information parafoveally along the reading direction despite age-related encoding difficulties.

To summarize, previous studies are equivocal about the efficiency of parafoveal processing in older readers. This topic is particularly interesting in Chinese, where characters are shown for their high visual complexity. For instance, Chang et al. [27] compared 131 written languages across four dimensions of visual complexity (i.e., perimetric complexity, number of disconnected components, number of connected points, number of simple features) and found that Chinese characters ranked highest across all dimensions. Traditional Chinese characters are also more complex than their simplified counterparts. Indeed, Gao and Kao [28] estimated that on average, simplified Chinese characters have 22.5% fewer strokes than traditional characters. Such high visual complexity may impose greater challenges in parafoveal processing, particularly in traditional Chinese older readers. The goal of this study is to further investigate the issue by examining whether age modulates the type of linguistic information (e.g., lexical and semantic) extractable in the parafovea.

### 1.3. Boundary Paradigm and Preview Benefit

The gaze-contingent boundary paradigm [6] is a key method for investigating the type of information available in parafoveal vision during sentence reading. In the boundary paradigm, participants are instructed to read sentences normally for comprehension. Unknown to the participants, an invisible boundary is placed just before a critical target word. Before a reader’s gaze crosses this boundary, the reader is shown either a valid (identical) or invalid (e.g., nonword or unrelated word) preview of the target. Once the reader’s gaze has crossed the boundary, the preview is instantly replaced with the target. Because this change occurs during a saccade, when vision is suppressed, readers typically remain unaware of both the preview identity and the display change. By measuring eye movement indices on the target when it is later fixated in different preview conditions, researchers have consistently observed facilitated reading performance, such as shorter fixation durations and fewer refixations, in valid compared to invalid preview conditions (see [1], for a review). This facilitation is known as the parafoveal preview benefit and is not limited to identical previews. It can also be observed with orthographically and phonologically related previews (e.g., [29,30,31]). Furthermore, while initial studies suggested a lack of semantic parafoveal preview benefit in English [32,33], subsequent research in Chinese has consistently demonstrated semantic, morphological, and syntactic preview benefits, arguably due to the logographic nature of the language (e.g., [34,35,36,37,38,39,40,41]). Semantic and syntactic preview benefits have also been observed in languages with regular orthography–phonology correspondence, such as German [42,43] and Korean [44,45], because fast access to phonology likely facilitates early access to semantic and syntactic information. In contrast, although semantic preview benefits have also been reported in English recently, they tend to occur only under specific conditions, such as when the preview word has a strong semantic association with the target (e.g., [46]) or when the sentence provides high contextual constraint (e.g., [47]).

Using the boundary paradigm [6], Rayner et al. [48] examined how older and younger readers respond to identical and unrelated previews during sentence reading. Both age groups demonstrated a parafoveal preview benefit in first fixation duration (FFD: duration of the first fixation on a word irrespective of the number of fixations). However, only younger readers showed a preview benefit in gaze duration (GD: the cumulative duration of all fixations during the first pass reading of the word). Different fixation measures provide an estimation of the time course of an effect: experimental effects that appear in FFD are considered to arise at an early temporal stage, earlier than those that emerge only in GD when the target word is refixated on, which are in turn earlier than those shown only in total reading time (TRT: sum of all fixations on a word, including regressive fixations) as a second-pass reading measure, reflecting a later processing stage [49,50]. As such, Rayner et al.’s [48] results indicate a more transient effect in older readers.

In another study, He et al. (2021) demonstrated that older readers of Chinese could extract parafoveal information from the second upcoming word to the right of the current fixation (the *N* + 2 position), similar to findings with German readers [24]. However, for older but not young readers, the *N* + 2 preview benefit was restricted to the condition where *N* + 1 was a valid preview. Payne and Stine-Morrow [51] compared the size of preview benefits when the preview (identical vs. nonword) was placed within a sentence or at the start of a clause/sentence. Given that resource-demanding wrap-up processing occurs at the end of a clause or sentence, the increased foveal load at these positions was expected to reduce the cognitive resources available for processing the preview, resulting in smaller preview benefits, particularly for older readers who have less resources overall. Results supported this prediction. Collectively, these findings suggest that parafoveal processing in older readers may be more constrained compared to younger readers.

While most previous studies evaluated preview benefits by comparing identical and nonword previews, a few have delved deeper into the types of linguistic information accessible to older readers in parafoveal vision. For instance, in a study of English readers, Choi et al. [52] used invalid previews that were orthographically similar to the identical previews (e.g., “livor” for “liver” and “heant” for “heart”). If older readers were unable to extract fine-grained orthographic information from the parafovea, they might not differentiate between valid and invalid preview conditions, potentially misreading invalid previews as valid ones. Contrary to this possibility, the study showed that older readers demonstrated preview benefits that were largely comparable to those of younger readers (see Table A2 in [52] for the absence of a preview × age interaction), indicating that older readers can obtain orthographic information in parafoveal vision. On the other hand, a somewhat different pattern emerged in Veldre et al. [13]. In their Experiment 1, a target word (e.g., rats) was paired with an identical preview, an orthographically similar preview (e.g., rate), a plausible continuation preview (e.g., junk), and an unrelated preview (e.g., sigh). The younger readers showed stronger identical and orthographic preview benefits than the older readers. However, the two groups exhibited comparable preview benefits in the plausible condition, suggesting that age-related modulation of the efficiency of parafoveal processing may depend on the type of linguistic information under consideration.

### 1.4. The Present Study

Existing findings about age-related differences in parafoveal preview remain inconclusive. Moreover, previous studies mainly examined identical preview and only occasionally included partial preview. An important contribution of the present study was to compare the effects of identical and different partial previews more systematically. Specifically, we compared the strength of identical, orthographic, phonological, and semantic parafoveal preview benefits (against an unrelated preview) between younger and older readers of traditional Chinese using the gaze-contingent boundary paradigm. Uncovering the types of information available to older readers in parafoveal vision is essential to extend eye-movement control models of reading (e.g., E-Z Reader and SWIFT) to different developmental groups. Given that prior research suggests that older readers in English can also extract linguistic information parafoveally [51,52], though with reduced efficiency (e.g., [13,48]), the present study predicts a similar reduction in the strength of the identical preview benefits among older Chinese readers. This prediction also reflects the cognitive decline in older adults [8]. On the other hand, age-related modulation on partial preview benefits remains under-explored. However, given the proposal that older readers have preserved knowledge and are able to use prior knowledge in reading [9], we expect relatively preserved semantic preview benefits in older Chinese readers. In contrast, they may exhibit smaller orthographic and phonological preview benefits compared to younger readers.

## 2. Method

### 2.1. Participants

Seventy older readers and 70 younger readers were recruited from the community through advertisement at community centers and online forums or through referral. Given that no prior studies have examined the age-related effects of orthographic, phonological and semantic preview benefits, the sample size was determined by referencing studies that examined identical preview benefits. For instance, Veldre et al. [13] had 65 older readers and 80 sentences in each experiment, while He et al. [53] had 44 older readers, 44 younger readers, and 60 sentences. The current sample size is comparable to these studies. All participants were born and raised in Hong Kong. They received formal education in Hong Kong and have never resided in other countries for more than one year. They were native Cantonese speakers and read traditional Chinese. None of them reported a history of psychological or neurological disorders. All older readers were above 60 years old at the time of testing (*M_age_* = 63.97 and *SD_age_* = 3.13), and they displayed normal visual acuity after wearing glasses (20/20 on the Snellen chart) (Older readers’ visual acuity was assessed in a screening test involving 134 participants. Those who did not meet the 20/20 criterion were not invited for the eye-tracking experiment.). Five older readers and two younger readers were discarded from further analyses because of low accuracy in answering the comprehension questions (<70%) in the sentence reading experiment. Table 1 shows the personal characteristics of the remaining 133 participants.

Due to changes in curriculum and education reform throughout the last two decades in Hong Kong (For instance, the years of free education extended from nine years to 12 years in 2008. Similarly, there were seven years of secondary school and three years of university education before 2012, which was then changed to six years of secondary and four years of university.), it is difficult to match old and young readers simply by the education level they attained. Therefore, several performance-based measures and self-reported ratings were used to evaluate the Chinese proficiency level of the participants. Firstly, LexCHI [54], which asks participants to identify all real words in a list of 60 two-character items (40 real words and 20 nonwords), was adopted. Secondly, given that the LexCHI test was originally designed for L2 Chinese users, the items tend to be too easy for native users, resulting in ceiling effects that may hinder the discriminative power of the test. To increase the test difficulty, Tsang et al. (preprint) (Available at OSF https://osf.io/j5uaq/ (accessed on 1 December 2025)) modified LexCHI by including items with a lexical decision accuracy of 65% to 85% in MELD-CH [55]. The modified LexCHI contains 60 words and 30 nonwords. The test score was shown to have a larger variation among native Chinese readers and to correlate with the self-rated reading proficiency and comprehension accuracy in Tsang et al. (preprint). The third performance-based test was a vocabulary test developed for the MELD-CH project. The test originally contained 24 multiple-choice questions. In each question, a Chinese word is shown and participants need to pick the correct definition of the word from four alternatives. After discarding four items that displayed low or negative item–rest correlations, the remaining items loaded on a single factor and the test score correlated significantly with lexical decision performance, LexCHI, self-rated Chinese proficiency, and Chinese subject score in public examination [56]. The revised 20-item version was used in this study. Finally, participants were asked to self-report the estimated number of hours of Chinese reading per week and their perceived Chinese proficiency in reading, listening, speaking, and writing on a 10-point scale (higher score = more proficient). Note that nine older readers and two younger readers did not complete the performance-based tests because of the time constraints. Nevertheless, they were included in the eye-movement analyses as they came from the same community sample and their self-reported proficiency was similar to other participants.

The independent sample *t*-test showed that old readers reported spending significantly more time on Chinese reading per week than young readers (*t*(131) = 2.41, *p* < 0.01), which was due to the fact that most younger readers were students or office workers that needed to handle English documents frequently. The older readers also displayed marginally higher vocabulary score (*t*(131) = 1.97, *p* = 0.052), but the two groups exhibited a similar level of Chinese proficiency in the two LexCHI tests and self-reported indices (all *p*s > 0.1). They also attained high and comparable levels (88.39% and 89.71%) of comprehension performance in the eye-tracking experiment (*p* > 0.2). Therefore, it is believed that the two groups had a similar level of Chinese proficiency, and any differences in the eye movement behaviors during reading cannot be attributed to an unmatched proficiency level.

### 2.2. Materials

One hundred and thirty sentences were selected from a Chinese sentence corpus [57]. For each sentence, a two-character word that was situated at least five characters away from the beginning and the end of the sentence was selected as the critical target. The target word was not predictable from the preceding context, as shown by a low average cloze probability (3.35%; range = 0% to 30%), which was obtained by asking 20 university students in Hong Kong to complete the sentences (shown up to the character before the critical targets) with the first words that came to their mind. The cloze probability for the preview characters in the semantic condition was 0.46%, while those in the other conditions were all 0% (Although the cloze probability of the semantic preview condition was below 1%, it was significantly different from the other conditions (*F*(3387) = 6.24, *p* < 0.001). However, removing items with non-zero cloze probabilities did not change the pattern of results (supplementary analyses available in OSF). Therefore, the reported results were based on the analyses of all items.).

The different preview conditions were then constructed by manipulating the first character of the critical target word, as shown in the example (Table 2). Specifically, the identical preview was the original character. The orthographic preview was a radical-sharing character without phonological overlap with the target, while the phonological preview was a homophonic character that was visually different. The semantic preview was a meaning-related character. A semantic relatedness rating between the original character and each of the non-identical preview characters was provided by 25 university students in Hong Kong on a 6-point Likert scale (higher score = more strongly related). The semantic preview characters were rated as more strongly related to the original characters than other preview characters (all *p*s < 0.001). There were no differences in semantic relatedness between the other preview conditions (all *p*s > 0.15). Finally, the unrelated preview characters were unrelated to the original character in orthography, phonology, and semantics. The characters in the different preview conditions were also matched on log-transformed number of strokes and character frequency (all *p*s = 1.00) retrieved from the “Chinese character database: With word-formations” [58].

### 2.3. Procedure

Potential participants first completed a simple online screening survey (with informed consent) that inquired their demographic information, language background and self-reported Chinese proficiency level. Those that met the inclusion criteria described above were then individually contacted and invited to participate in the eye-tracking experiment, which was conducted individually at a sound-attenuated laboratory. Participants provided another informed consent before participating in the eye-tracking experiment. The experimental protocol was approved by the Research Ethics Committee at Hong Kong Baptist University.

Participants were provided with information about the eye-tracking technique, including the need to use a chin and forehead rest to minimize head movement for more accurate tracking. The eye tracker model was Eyelink Portable Duo (SR Research, Ottawa, Canada), running in a head-stabilized mode. The sampling rate was 500 Hz at the head-stabilized tracking mode. A laptop computer controlled the eye tracker, which was connected to a desktop computer for stimulus presentation. The stimuli were presented on a 25-inch LDC monitor (Philips, Hong Kong, China; resolution = 1920 × 1080 pixels; refresh rate = 240 Hz). Participants were seated at about 60 cm from the monitor. At this distance, each Chinese character subtended about 1.3° of visual angle. This stimulus size is slightly larger than previous studies (1° in [54]) but is within the normal range of 0.3° to 2.0° [59]. Viewing was binocular, but recording was performed on the right eye only. Participants completed the 9-point calibration and validation procedure before reading the sentences. Using the default settings of the eye tracker, the procedure ensured that the average error was smaller than 0.5°.

Stimulus presentation and response recording were controlled by Experiment Builder (SR Research, Ottawa, ON, Canada). In each trial, a fixation dot first appeared on the left side of the screen for drift correction. If the participants’ fixation did not fall on the fixation dot, a recalibration would be performed. Stable fixation on the fixation dot would trigger presentation of the sentence, with the first character of the sentence replacing the fixation dot. The sentence was presented horizontally (from left to right) in one line and in black color on a light gray background. For the gaze-contingent display change, an invisible vertical boundary was placed just before the critical character. Before the eyes crossed the boundary, the preview character was displayed. When the eyes crossed the boundary, the preview character switched to the correct character. Due to the suppression of visual information intake during a saccadic movement, participants were typically unaware of the display change. Participants were instructed to read the sentence for comprehension, but they were not informed about the presence of the invisible boundary or display change. When participants finished reading a sentence, they needed to fixate on a dot at the lower right corner of the screen and pressed the spacebar on the keyboard to indicate completion of the trial and initiate the next trial. To ensure attentive reading, a true or false comprehension question appeared after 28 sentences. Participants needed to indicate their responses by pressing keys on the keyboard. In these cases, the next trial began after completion of the comprehension questions.

Participants were given 12 practice trials with four comprehension questions before the main experiment. The practice trials were repeated if the participants requested clarification of the procedure. Throughout the experiment, a chin and forehead rest was used to minimize head movement. Participants were also requested to avoid body movement as much as possible when reading the sentences, but they could take a break at the beginning of any trial when the drift correction dot was shown (because the trial would not begin if they did not fixate on that dot). If participants took a rest, a recalibration would be performed before moving on. Recalibration was also performed whenever the experimenter considered it necessary. After completing the main reading experiment, participants completed the LexCHI, modified LexCHI, and vocabulary tests. As aforementioned, 11 participants (2 younger readers and 9 older readers) did not complete these tests because of the time constraints. The whole experiment lasted for 60 to 75 min.

### 2.4. Analyses

Data for one sentence was discarded because of a misplaced boundary. For the remaining data, the critical region of interest was the entire two-character target word that contained the manipulated preview character. Four eye movement indices were retrieved. Initial skipping probability (SP: the chance that the target word was not fixated in the first pass), first fixation duration (FFD), gaze duration (GD), and total reading time (TRT). Data was screened using customed MATLAB (version 2021a)script, following previously used protocol [36,60]. First, trials containing missing samples, tracker errors or blinks were removed. Second, FFDs shorter than 60 ms or longer than 800 ms and GDs longer than 1000 ms were removed (older readers: *n* = 82, 1.7%, and young readers: *n* = 64, 1.5%). Third, trials were removed when there were regressions from pretarget or target words (older readers: *n* = 523, 10.9%, and young readers: *n* = 400, 9.7%) because they may reflect incomplete parafoveal processing during preview or incomplete foveal processing of the target words, as a regression from a word may occur before lexical processing of the word is completed (e.g., [61]). Finally, specific to the gaze-contingent boundary paradigm, trials in which display changes were triggered improperly during fixations were excluded (older readers: *n* = 221, 4.6%, and young readers: *n* = 257, 6.2%) because readers may perceive the display change. In the end, there were 7492 valid data points, retaining 4031 (83.9%) and 3461 (83.6%) of fixated target words from the older and the younger readers, respectively. A power analysis was conducted using the simr package (version 1.0.8; [62]). We used data from the first 10 participants (five younger and five older readers) to estimate the effect sizes and simulate statistical power. The simulation results indicated that obtaining 500 observations per condition, or the total sample size of 130 participants, would yield statistical powers above the conventional 80% threshold for most effects. The only exception was the age-group effect in TRT, which reached a power of 71%. Overall, the analyses reported below can therefore be considered sufficiently powered.

Analyses were performed using linear mixed-effects models (for the time-based indices of FFD, GD, and TRT) and generalized linear-mixed models with a binomial distribution (for the proportion-based index SP). The models were estimated using the lme4 package (version 2.0-1 [63]) implemented on the R platform (version 4.5.3). The *p*-values for individual parameter estimates were obtained based on Satterthwaite approximation using the lmerTest package (version 3.2-1; [64]). The four eye movement indices were the dependent variables of interest. The time-based indices were log-transformed. The fixed factors were age group (older vs. younger readers) and preview condition (identical, orthographic, phonological, semantic vs. unrelated). Age group was sum contrast coded (−0.5 = old readers; 0.5 = young readers) such that the intercept would reflect the grand mean. The preview condition was treatment contrast coded with the unrelated preview as the reference. This coding scheme directly reflects the preview benefits obtained in the identical condition and each partial sharing condition, as compared to the unrelated baseline. The random structure was initially kept as maximal, which contained the by-participant and by-item random intercepts and the by-participant random slope of the preview condition and the by-item random slopes of age group and condition. However, because of convergence problems, and following the parsimonious LMM principle [63,65], random effects that had low variances were removed one by one until the models converged. For SP, the final model that converged was the intercept-only model. For FFD, it contains the by-participant random slope of the identical vs. unrelated contrast. For GD, it contains the by-participant random slope of the identical vs. unrelated contrast and orthographic vs. unrelated contrast. For TRT, the final model contained all by-participant random slopes except the phonological vs. unrelated contrast. The data, R script, and detailed output of the analyses are available in OSF (see data availability statement). The extraction of data and corresponding variable names in the data file follow those in the Beijing Sentence Corpus [66].

## 3. Results

The remaining participants correctly answered at least 70% of the comprehension questions and the comprehension rate did not differ statistically between the older readers (M = 88.4%, SD = 6.7%) and the young readers (M = 89.7%, SD = 5.6%), *t*(131) = 1.2 and *p* > 0.1. Table 3 shows the descriptives of the four eye movement indices in each condition. Notably, younger readers appear to have much higher skipping rates than older readers, which agrees with previous Chinese reading studies [17,67], but is inconsistent with the risky reading strategy observed in older English readers [4]. The canonical preview benefit, which is the difference in processing identical previews as compared to unrelated previews, can be seen in all duration-based measures in both age groups. However, the size of the identical preview benefit appears to be generally smaller among the older readers, except for TRT. In addition, partial preview in the orthographic, phonological, and semantic conditions seems to be helpful only to young readers in the early stages of processing reflected by the FFD and GD. These observations are statistically confirmed. A summary of the results is provided in Table 4. Full scripts and results of the linear mixed-effects models are available in OSF.

**Figure 1:**
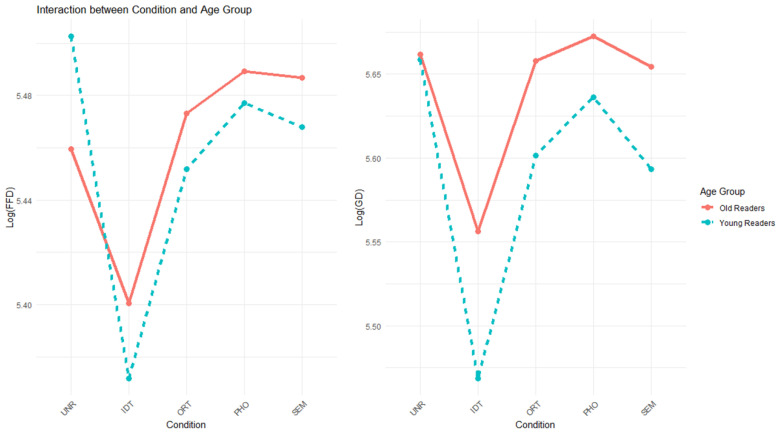
Interactions between condition and age group in FFD (**left**) and GD (**right**) based on the model predictions.

Consistent with the observations, there was a significant age group effect in SP (*b* = 1.30, *SE* = 0.26, *z* = 4.98, *p* < 0.001), reflecting an overall lower skipping probability among older readers. However, the SP did not differ across the preview conditions. In contrast, the identical preview led to a shorter FFD than the unrelated preview condition (*b* = −0.097, *SE* = 0.013, *t* = −7.63, *p* < 0.001), which was further qualified by a significant interaction with age group (*b* = −0.075, *SE* = 0.025, *t* = −2.96, *p* < 0.01). Moreover, age group also modulated the other condition contrasts. To clarify the origin of these interactions, further analyses were conducted within each age group. The results are presented in Table 5 and Figure 1.

Table 5 shows that while younger readers displayed shorter FFDs in both identical (*b* = −0.14, *SE* = 0.018, *t* = −7.34, *p* < 0.001) and orthographic (*b* = −0.050, *SE* = 0.019, *t* = −2.69, *p* < 0.01) preview conditions, as compared to the unrelated condition, older readers only benefited from identical previews (*b* = −0.060, *SE* = 0.018, *t* = −3.33, *p* < 0.001). Moreover, as shown in Figure 1 (left), the size of the identical preview benefit was smaller among older readers.

A similar pattern was observed for the GD, where the identical preview (*b* = −0.15, *SE* = 0.017, *t* = −8.92, *p* < 0.001) and semantic preview (*b* = −0.035, *SE* = 0.016, *t* = −2.17, *p* < 0.05) benefits were significant overall and were both moderated by age group (*b* = −0.092, *SE* = 0.034, *t* = −2.70, *p* < 0.01 and *b* = −0.066, *SE* = 0.032, *t* = −2.05, *p* < 0.05, respectively). Further analyses in each age group (Table 5) again suggested that older readers only benefited from identical previews (*b* = −0.11, *SE* = 0.022, *t* = −4.86, *p* < 0.001). In contrast, younger readers showed significantly shorter GDs in the identical (*b* = −0.20, *SE* = 0.023, *t* = −8.67, *p* < 0.001), orthographic (*b* = −0.056, *SE* = 0.023, *t* = −2.40, *p* < 0.05) and semantic preview conditions (*b* = −0.069, *SE* = 0.023, *t* = −2.98, *p* < 0.01), a pattern similar to previous studies with university student participants [31,35].

A somewhat different pattern emerged in TRT, where all types of previews led to a shorter TRT than the unrelated baseline (identical: *b* = −0.26, *SE* = 0.018, *t* = −14.82, *p* < 0.001; orthographic: *b* = −0.052, *SE* = 0.018, *t* = −2.84, *p* < 0.01; phonological: *b* = −0.038, *SE* = 0.018, *t* = −2.19, *p* < 0.05; semantic: *b* = −0.053, *SE* = 0.018, *t* = −3.00, *p* < 0.01). The magnitude of the preview benefits on TRT was statistically comparable between older and younger readers, as evidenced by the lack of significant interactions with age group (all *p*s > 0.26). Finally, no significant main effects of age were observed in all fixation time measures, a pattern similar to that reported by Rayner et al. [48] and Choi et al. [52].

## 4. Discussion

This study investigates age-related differences in parafoveal preview benefits during Chinese sentence reading. Based on previous studies, we have predicted that older readers would exhibit weaker preview benefits for identical, orthographic, and phonological previews, but they might display similar benefits for semantic preview. The results are partially consistent with these predictions. Specifically, we showed that age interacted with preview conditions in first-pass reading indices (FFD and GD), suggesting differential preview benefits between younger and older readers in early processing. Further analyses revealed that younger readers benefited from identical and orthographic previews (against the unrelated baseline) in FFD, and from identical, orthographic and semantic previews in GD. In contrast, for older readers, the preview benefits were smaller and restricted to the identical condition. We observed no evidence that older readers displayed intact semantic preview in early eye-movement measures. Interestingly, the size of the preview benefits became comparable between younger and older readers in TRT, which reflects a relatively later stage of reading.

### 4.1. Age-Related Differences in Parafoveal Preview Benefits

In this study, preview conditions failed to influence initial skipping rates, which aligns with Rayner et al. [48]. Among younger readers, the parafoveal preview benefits largely corroborate previous research in Chinese [35]. Moreover, orthographic and semantic preview benefits emerged relatively early in FFD and GD, respectively, occurring earlier than phonological preview benefits in TRT. This pattern generally concurs with previous findings, suggesting relatively later parafoveal phonological activation than semantic activation in Chinese [68,69].

Older readers exhibited reduced preview benefits at early processing stages during reading, while they displayed preview benefits comparable to younger readers in TRT. We interpret this pattern as indicating their ability to extract various linguistic information from the parafovea, although the efficiency of parafoveal processing is reduced due to age-related decline in visual function [7]. Alternatively, one may argue that the effects in TRT only reflect older readers’ better ability in late-stage recovery (presumably attributable to their life-long reading experiences), and do not indicate any capacity to extract the partial parafoveal information. However, if this was the case, recovery should be equally effective in the unrelated condition, resulting in no differences across conditions at all. It should be noted that by the time readers’ gazes crossed the invisible boundary, the preview characters would change to the correct ones. Therefore, the visual information that supported the differences in later stages had to be obtained during the first pass reading, even when the effects emerged later. In other words, we believe it is more reasonable to suggest that the preview effects in TRT reflect the continual internal processing of the previews without further bottom-up visual inputs.

The ability of older readers to extract parafoveal information in a less efficient way than younger readers suggests that older readers may have an intact perceptual span [15,21] which supports their parafoveal processing in the first place. This further strengthens the view that parafoveal processing depends not only on visual factors but also on attentional factors [24,26]. At the same time, the slower emergence of preview benefits also aligns with age-related decline in general processing speed [8]. This time factor indicates that it is important to consider the temporal progression of preview benefits to understand age-related changes in parafoveal processing. Parafoveal processing in older adults can be both preserved and weakened, depending on the temporal stage of processing.

A widely accepted explanation of parafoveal preview benefits attributes the effect to the trans-saccadic integration of linguistic information during parafoveal and foveal processing [6]. When an identical or a related preview is available parafoveally, it activates the relevant linguistic representations and provides a head start when the previewed target is fixated on, facilitating its recognition and reducing fixation duration. Yet, it is unclear how this integration depends on age. On the other hand, the integration process is analogous to priming in isolated word recognition, where prior exposure to related information speeds up processing. Considering the similarity, important insights may be gained from the substantial body of literature on isolated word recognition.

In word recognition, facilitative priming occurs when a briefly presented prime word activates its lexical representation, which in turn spreads activation to related representations, including the target word. This partially activated target can then reach the recognition threshold more rapidly or with less bottom-up input. Older readers typically exhibit preserved or even enhanced priming effects in isolated word recognition [70,71]. While some researchers took this as supporting intact spreading activation in older adults (e.g., [72]), alternative explanations have been proposed. According to Laver and Burke’s [70] transmission deficit hypothesis (TDH), this phenomenon can be explained by assuming that aging weakens the connections between lexical representations, thereby slowing the transmission of spreading activation. Consequently, more time is required to build up the level of activation necessary for priming to occur. At the same time, older adults’ extensive language experience may result in more connections within the lexical network, allowing the summation of spreading activation to compensate for the weakened connections, provided that enough time is allowed for priming to accumulate. This is typically the case, as older adults generally have slower response times. As a result, older adults can demonstrate a priming effect comparable to that of younger adults.

Although the TDH was initially proposed to explain semantic priming in isolated word processing, similar mechanisms can be applied to the current findings of weaker identical and absent partial preview benefits in FFD and GD, alongside intact preview benefits in TRT. Specifically, older readers can successfully process the parafoveal preview word despite reduced visual acuity. The preview word’s representation begins to activate, but the process of spreading activation and integration between parafoveal and foveal representations may be slower in older adults. This slower processing reduces the strength of the preview benefits in early eye movement measures when the preview target is fixated. Identical previews are relatively more effective in producing preview benefits because they provide a boost across all linguistic dimensions (orthographic, phonological, and semantic). However, partial previews remain too weak to facilitate processing until the later stages, as reflected by TRT, where older readers’ extended processing of the preview words over time allows the preview benefits to accumulate to a level comparable to those in younger readers, as in the case of isolated word priming. Therefore, the preview benefits become comparable in size between older and younger readers in TRT, even though they are initially weaker in FFD and GD.

The idea of weaker connections between lexical representations in older adults aligns with recent semantic network analyses across the lifespan [73,74], which indicate that the network becomes sparser and exhibits weaker connectivity between items as individuals get old. The weaker spreading activation in older adults may also be rooted in their reduced efficiency of brain networks [75].

Most existing models of eye movement control in reading contain parameters related to age-related declines in visual acuity and processing speed [76,77]. In contrast, it is less clear how the changes in memory and semantic networks due to a lifetime of reading experience will affect reading. In principle, they may affect the speed of familiarity check and lexical access in E-Z Reader or modulate the shape and extent of attention gradient in SWIFT, but formal simulation is needed. In addition, Snell et al. [5] proposed that to better simulate reading behaviors, an explicit word recognition module should be incorporated into eye movement models. The present study, together with recent reports of age-related changes ([3,20] for review), suggests some important constraints in developing aging-sensitive models. For instance, under the framework of E-Z Reader, the time of fixation on a word depends on both word recognition and oculomotor systems. The general cognitive slowing due to aging may affect these components differently. It may be the case that word recognition is impaired more due to inefficient spreading activation as described above, while the oculomotor control is relatively intact because it is a highly practiced procedural behavior. Then, older readers may move their eyes to the next words without sufficient processing of the current one, leading to more regressions with the previews continuing to exert their influences at a later time point. Future improvements in reading models can more explicitly describe how the model architecture can flexibly account for the complex interplay between cognitive decline and accumulated experience. More generally, formal simulations will be needed to clarify the complex interplay between various age-related factors (e.g., reduced visual acuity and processing speed together with increased size of the lexicon and prior reading experience).

### 4.2. Reading Strategies in Chinese Older Readers

In this study, older readers were generally less likely to skip previewed words compared to younger readers. This lower skipping rate is consistent with other eye-tracking studies in Chinese [17,67], but contrasts with findings from alphabetic scripts [4,19]. A recent meta-analysis by Zhang et al. [20] indeed revealed that older readers tend to skip words more frequently only when reading alphabetic scripts. Therefore, the data suggests that older Chinese readers do not adopt a risky reading strategy like readers of alphabetic scripts. The present results extend previous findings by showing that older readers of Chinese continue to skip less regardless of the types of information available in the parafovea, suggesting that the nature and availability of parafoveal linguistic information does not modulate the skipping rate in older Chinese readers.

The reasons why older Chinese readers do not adopt this risky strategy remain unclear. Zhang et al. [20] proposed that the higher visual demands associated with the visual complexity of Chinese characters and the absence of explicit word boundaries may be contributing factors. This idea is supported by a recent study showing reduced GD and TRT among Chinese readers, especially the older ones, when word boundary cues are provided [16]. Similarly, McGowan and Reichle [78] suggested that the visual complexity of Chinese characters may hinder older readers’ abilities to use partial parafoveal information to guess upcoming words. The present results clarify this claim by showing that older readers are indeed capable of extracting partial parafoveal information. Their limitation seems to be primarily due to a slower speed in utilizing this information (in spreading activation, as discussed above), rather than a complete inability to use it (The present study used a relatively large font size (1.3°) to ensure readability for older readers. Although it is within the range recommended by Xu and Jordan [59], it might inadvertently increase the distance of the preview character from the fixation point, reducing parafoveal processing efficiency in Chinese [39] and leading to slower activation of the preview characters. Nonetheless, it should again be noted that the preview characters were processed sufficiently extensively in the first pass to produce intact preview benefits in TRT.).

The lower skipping rate observed in older Chinese readers may also relate to cross-language differences in the overall predictability of words in texts. Afterall, if the next word is not sufficiently predictable, a strategy that prioritizes guessing will not be optimal. Although experimental manipulations of predictability showed that older Chinese readers are sensitive to this variable in reading (e.g., [20,67]), certain features of the Chinese language may discourage a universal guessing-based strategy. For example, over 40% of Chinese characters are homonyms [79], compared to only 7.4% of English words being homonyms [80] (Boundary paradigm experiments in Chinese typically used character preview, while those in English used word preview. Therefore, the comparison here is between Chinese characters and English words. Chinese words are less ambiguous than characters.). Homonymous characters are represented differently [81] and require disambiguation during word recognition, making their processing more complicated [55,82]. When older readers encounter such homonymous characters parafoveally, they may lack the cognitive resources needed to clarify the correct meanings, making accurate guesses difficult.

Finally, it is worth considering whether the lower skipping rates observed in older Chinese readers indicate a “conservative” reading strategy [78]. Early research [4,19] proposed a risky reading strategy for older readers due to the seemingly contradictory findings of longer reading times paired with higher skipping rates. The rationale was that if cognitive processing is slowed by age-related declines, older readers should also skip words less frequently. Thus, a compensatory strategic processing mechanism was needed to explain this contradiction. In contrast, older readers in Chinese exhibit both slower reading speeds and lower skipping rates than younger readers, which can be explained simply as quantitative differences in processing speed and spreading activation efficiency. Therefore, we suggest that a specific reading strategy is not needed to account for the reading behaviors of older Chinese readers.

## 5. Limitations and Conclusions

To the best of our knowledge, this study is the first to systematically investigate age-related changes in different types of partial preview benefits (orthographic, phonological and semantic). The results indicate weakened parafoveal previews in early processing stages for older readers, as indexed by FFT and GD, while the preview benefits in later processing stages, reflected in TRT, were comparable to those of younger readers. We suggest that this pattern can be explained by age-related declines in the speed of spreading activation and integration across parafoveal and foveal information. Despite these contributions to understanding age-related differences in parafoveal processing, several limitations should be addressed in future studies. Firstly, although a power analysis revealed that the present study has over 80% power, the absence of preview benefit in the older readers group is still a null effect that should be interpreted cautiously. Future research is needed to replicate the findings with more participants and items. Secondly, the older participants in this study were relatively young (mean age = 64), well-educated (more than 40% having university-level education or higher), and had preserved vision, which may have contributed to their good performance. Future research should examine changes in older old participants aged 75 and above. Moreover, we have only measured the vocabulary knowledge of the participants. Other cognitive assessments may be needed to provide a more comprehensive understanding of participants’ cognitive profiles. More generally, it will be important to explore how individual differences may influence age-related effects on cognitive functions [83], including reading. Thirdly, the materials used were in traditional Chinese. Given the differences in the visual complexity of simplified and traditional Chinese characters [28] and findings that there are both similarities and differences in processing traditional and simplified Chinese [60,66,84], it is not immediately clear whether the present results are generalizable to simplified Chinese. Finally, the proposal of less efficient spreading activation is admittedly speculative. In particular, we did not directly measure the efficiency of spreading activation, and alternative explanations are possible. For instance, it may be the case that preview benefits are best reflected in early eye movement measures, and late measures like total reading times actually reflect downstream processes like integration or re-analysis that may be unaffected by parafoveal preview. These alternatives need to be tested empirically in future studies.

## Figures and Tables

**Table 1:** Participant characteristics.

	Old Readers	Young Readers
Final sample size	65	68
Age	63.97 (3.13)	21.04 (2.48)
Gender	Male: 29 Female: 36	Male: 20 Female:48
Education level	Secondary or below: 36 University: 16 Postgraduate: 13	Secondary: 45 University: 17 Postgraduate: 6
Comprehension accuracy in the eye-tracking experiment	88.39% (6.71)	89.71% (5.67)
LexCHI	91.23 (13.19)	92.46 (14.40)
Modified LexCHI	74.82 (17.04)	70.81 (15.69)
Vocabulary test accuracy	54.29 (19.29)	47.88 (16.71)
Estimated hours of Chinese reading per week	10.07 (9.42)	6.39 (8.21)
Self-rated Chinese proficiency in listening	7.75 (1.12)	7.66 (1.44)
Self-rated Chinese proficiency in speaking	7.40 (1.13)	7.21 (1.41)
Self-rated Chinese proficiency in reading	7.62 (1.16)	7.57 (1.51)
Self-rated Chinese proficiency in writing	6.75 (1.26)	7.00 (1.48)

Note: LexCHI and modified LexCHI were scored using the normalized Ghent formula, which penalizes false alarm to nonwords (see [54]). Nine older readers and two younger readers did not complete the performance-based tests because of time constraints.

**Table 2:** Stimulus characteristics (standard deviations in parentheses).

Example Sentence: 一個良好的候車環境可以緩解人的緊張心理. Translation: A Good Waiting Environment Can Alleviate People’s Anxiety. Critical Character: 環/waan4/ring (of the Two-Character Word 環境 Environment).					
	Identical	Ortho	Phono	Semantic	Unrelated
Character	環	瑪	頑	圈	舞
Pronunciation	/waan4/	/maa5/	/waan4/	/hyun1/	/mou5/
Meaning	ring	agate	stubborn	circle	dance
Frequency (log)	3.38 (0.60)	3.36 (0.67)	3.44 (0.62)	3.40 (0.70)	3.40 (0.58)
Stroke number	10.72 (3.74)	10.75 (3.75)	10.22 (3.85)	10.30 (3.95)	10.61 (3.93)
Cloze probability	3.35 (6.21)	0 (-)	0 (-)	0.46 (2.11)	0 (-)
Sem relatedness	-	1.68 (0.41)	1.68 (0.31)	4.85 (0.60)	1.55 (0.41)

Note: Ortho = orthographic; phono = phonological; sem relatedness = semantic relatedness with the critical character as rated on a 6-point Likert scale (6 = highly related). Given that the cloze probabilities in the ortho, phono, and unrelated conditions are 0%, there are no standard deviation values.

**Table 3:** Fixation measures as a function of preview condition and age group.

	Identical	Orthographic	Phonological	Semantic	Unrelated
Old SP	0.197	0.194	0.183	0.176	0.163
	(0.0138)	(0.0139)	(0.0135)	(0.0135)	(0.0132)
	[0.170, 0.224]	[0.167, 0.221]	[0.156, 0.209]	[0.150, 0.203]	[0.137, 0.189]
Young SP	0.333	0.325	0.312	0.320	0.328
	(0.0176)	(0.0179)	(0.0175)	(0.0177)	(0.0182)
	[0.299, 0.368]	[0.289, 0.360]	[0.278, 0.347]	[0.285, 0.355]	[0.292, 0.364]
Old FFD	234	254	259	257	252
	(2.69)	(3.30)	(3.38)	(3.35)	(3.42)
	[229, 239]	[247, 260]	[252, 266]	[251, 264]	[245, 259]
Young FFD	230	249	255	253	263
	(3.36)	(3.66)	(3.63)	(3.69)	(4.04)
	[224, 237]	[242, 256]	[248, 262]	[246, 261]	[256, 271]
Old GD	287	322	327	319	327
	(4.77)	(5.79)	(5.75)	(5.60)	(6.19)
	[278, 296]	[310, 333]	[316, 338]	[308, 330]	[315, 339]
Young GD	263	304	312	299	319
	(4.83)	(5.97)	(5.71)	(5.64)	(5.99)
	[253, 272]	[292, 315]	[301, 323]	[288, 310]	[308, 331]
Old TRT	350	423	434	433	452
	(7.01)	(8.59)	(9.03)	(9.10)	(9.75)
	[336, 364]	[407, 440]	[417, 452]	[415, 451]	[433, 471]
Young TRT	333	407	409	415	435
	(7.27)	(9.96)	(8.68)	(10.30)	(10.20)
	[318, 347]	[388, 427]	[392, 426]	[394, 435]	[415, 455]

Note: SP = initial skipping probability; FFD = first fixation duration; GD = gaze duration; TRT = total reading time; FFD, GD, and TRT are in ms. Standard error of means (for FFD, GD, TRT) or standard error of proportions (for SP) in parentheses.

**Table 4:** Results of linear mixed-effects models (FFD, GD, and TRT) and generalized linear mixed-effects model (SP).

		SP			FFD			GD			TRT	
	*Estimate*	*S.E.*	*z*	*Estimate*	*S.E.*	*t*	*Estimate*	*S.E.*	*t*	*Estimate*	*S.E.*	*t*
Intercept	−1.412	0.142	−9.963	5.472	0.015	365.535	5.648	0.020	284.944	5.935	0.027	216.015
Age Group	1.297	0.261	4.975	0.029	0.029	0.992	−0.014	0.038	−0.368	−0.030	0.051	−0.586
Preview:												
Identical	0.183	0.098	1.870	−0.097	0.013	−7.628	−0.152	0.017	−8.917	−0.262	0.018	−14.823
Orthographic	0.061	0.099	0.613	−0.016	0.013	−1.258	−0.027	0.018	−1.556	−0.052	0.018	−2.842
Phonological	0.078	0.099	0.785	0.000	0.013	0.014	−0.005	0.016	−0.305	−0.038	0.018	−2.191
Semantic	0.024	0.100	0.235	−0.002	0.013	−0.195	−0.035	0.016	−2.168	−0.053	0.018	−2.874
Interaction:												
Identical × Age	−0.267	0.195	−1.364	−0.075	0.025	−2.955	−0.092	0.034	−2.703	−0.040	0.035	−1.127
Ortho × Age	−0.305	0.198	−1.536	−0.068	0.025	−2.710	−0.062	0.035	−1.753	−0.035	0.036	−0.959
Phono × Age	−0.242	0.199	−1.219	−0.055	0.025	−2.181	−0.027	0.032	−0.847	−0.007	0.035	−0.202
Semantic × Age	−0.125	0.200	−0.623	−0.062	0.025	−2.455	−0.066	0.032	−2.045	−0.041	0.037	−1.126

Note: SP = initial skipping probability; FFD = first fixation duration; GD = gaze duration; TRT = total reading time. FFD, GD, and TRT were log-transformed in the analyses. Treatment contrast coding was used for condition (unrelated condition as baseline). Sum contrast coding was used for age group. Full outputs including random effects are also available online in OSF. Significant effects are shaded. Dark shading represents *p* < 0.001; medium shading represents *p* < 0.01; light shading represents *p* < 0.05.

**Table 5:** Separate analyses of old and young readers in FFD and GD.

	FFD						GD					
		Old			Young			Old			Young	
	*Estimate*	*S.E.*	*t*	*Estimate*	*S.E.*	*t*	*Estimate*	*S.E.*	*t*	*Estimate*	*S.E.*	*t*
Intercept	5.457	0.020	270.049	5.487	0.022	253.033	5.654	0.028	203.803	5.642	0.027	208.220
Identical	−0.060	0.018	−3.328	−0.135	0.018	−7.343	−0.108	0.022	−4.857	−0.199	0.023	−8.672
Orthographic	0.018	0.017	1.063	−0.050	0.019	−2.693	0.003	0.023	0.140	−0.056	0.023	−2.403
Phonological	0.027	0.017	1.600	−0.028	0.019	−1.489	0.008	0.022	0.373	−0.019	0.023	−0.841
Semantic	0.028	0.017	1.664	−0.034	0.019	−1.843	−0.002	0.023	−0.100	−0.069	0.023	−2.983

Note: FFD = first fixation duration; GD = gaze duration. FFD and GD were log-transformed in the analyses. Treatment contrast coding was used for condition (unrelated condition as baseline). Full outputs including random effects are available online in OSF. Significant effects are shaded. Dark shading represents *p* < 0.001; medium shading represents *p* < 0.01; light shading represents *p* < 0.05.

## Data Availability

The original data presented in the study are openly available in OSF at https://osf.io/j5uaq/ (accessed on 1 December 2025).
